# Prolonged Cough in Pediatric Population First Line Care, Belgian Guidelines

**DOI:** 10.2174/1874306401711010054

**Published:** 2017-08-21

**Authors:** Sophie Leconte, Stéphanie Valentin, Estelle Dromelet, Michel De Jonghe

**Affiliations:** 1 Centre académique de médecine générale, Université catholique de Louvain, Bruxelles, Belgium; 2 Institut de Recherche santé et société, Université catholique de Louvain, Bruxelles, Belgium

**Keywords:** Cough, Primary care, Guideline, Child, Chronic, Subacute

## Abstract

**Background:**

The clinical approach to a prolonged cough, *i.e*. a cough lasting more than three weeks, is challenging for general practitioners as well for primary care pediatricians. What the recommended clinical approach in primary care is, how cough duration or cough characteristics impact the diagnosis, and what the efficiency and safety of antibiotics or symptomatic treatments are remain in question for primary care physicians.

**Objective:**

The last Belgian guidelines were published in 2006 and needed to be reviewed. Those background questions were used to conduct our guideline updating procedure.

**Methods:**

We systematically performed a pyramidal literature search between the periods 2006-2014 in order to write evidence based guidelines. The data of the literature was summarized, discussed by the authors, experts and the Belgian primary care guidelines committee. Recommendations were formulated and scored following the GRADE classification.

**Results:**

The consultation history as well as the physical examination should be directed towards searching for warning signs (GRADE 1B) and towards the common etiologies depending on cough duration (GRADE 2C). If the cough lasts for more than eight weeks, chest radiography and spirometry should be considered (GRADE 2C). An antibiotic is recommended for a prolonged wet cough (over eight weeks) if prolonged bacterial bronchitis is suspected (GRADE 1B). In the absence of clinical signs of a specific etiology of a cough, no drug can be recommended (GRADE 1B). For all cases, it is initially suggested to avoid irritants (GRADE 1C) as well as to take into account the concerns of parents and inform them about the natural development of a cough.

**Conclusions:**

More research is needed to provide evidence on the clinical pathway on prolonged cough for primary care. Cough duration of more than eight weeks and prolonged wet cough are the most useful cough characteristics. Regarding a specific cough treatment, no medication has proved any effect greater than placebo. Attention to environmental triggers and patient-centered care remain the keystones of interventions

## 1 INTRODUCTION

A prolonged cough is a common symptom in children. According to various studies of school children, between 5 and 10% of children experience a prolonged cough episode [1-4].

However, we know that not all patients with a health problem complain, and that only a certain percentage of them contact their general practitioner (GP) [5]. An even smaller percentage will consult a specialist. Two and a half per cent of general practice visits of children aged five to 15 years are due to a prolonged cough [6]. Retrospectively, between 10 and 25% of children have a history of prolonged cough mentioned in their medical records [6].

We will first address some definitions related to our subject and epidemiological issues.

### 1.1 Pathological Cough and Subjectivity of the Complaint

These recommendations do not address an illness but a symptom, as reported by the patient. This symptom, cough, is above all a reflex to protect the lungs. Studies have attempted to quantify this reflex in “healthy” children. It appears that healthy children cough on average 10 times a day [7]. In the definition of chronic cough, literature does not define a threshold in terms of frequency or intensity of the cough, so we will consider the threshold that parents complain of.

Different studies have found that the intensity of the cough as judged by parents did not correlate with an objective account of the impact on the quality of life [8]. For an acute cough, parental anxiety or the fact of living in a city are associated with a more frequent consultation rate [9].

Prolonged cough affects the quality of life of children and their parents. The main consequences are frustration at not being able to relieve the distress of the child, sleepless nights and finally the stress of the symptoms on the child [9, 10]. Anxiety can be caused by the risk of suffocation, lung damage or the risk of sudden death. Anxiety is greatest at night, when parents have less control over the situation and the child seems vulnerable [11]. This feeling of loss of control, and not being able to help or protect their child is something that forces parents to seek medical help [12].

The same complaint can therefore cover very different realities. The diagnosis spectrum could be affected by this first cough presentation and pattern. Taking into account these elements, we agree that, as with other complaints in general practice, a comprehensive approach is needed in the diagnostic process to better understand the reality of the complaint and its impact, and when considering the care plan it is necessary to take into account the anxiety and concerns of parents as well as this first clinical evaluation of the complaint.

### 1.2 Duration of Cough

In terms of the duration of the episode, various thresholds are used in the literature. There is no agreed definition of chronic or subacute cough for children or adults. Thresholds of what a prolonged cough is vary. A recent meta-analysis summarized prospective studies evaluating the duration of a cough after an acute episode. It appears that 50% of coughs are resolved on day 10 and 90% on day 25 [13]. In view of the natural evolution of an acute cough, we can consider a first threshold may be set after four weeks of coughing. Therefore, the recommendations define an acute cough within that time.

### 1.3 Etiology-Based Cough Duration

Epidemiological studies have shown clinicians that different etiologies exist regarding the cough duration, and the probability of a particular cough etiology occurring varies depending on the cough duration

*A cough lasting up to four weeks*, *i.e*. an acute cough, is most often due to a viral respiratory infection that is waning normally [13, 14]. *For a cough lasting between four and eight weeks*, *i.e*. a prolonged cough, prolonged infections (Mycoplasma pneumoniae, Bordetella pertussis, adenoviruses) or recurring infections are the most common causes [15-17].

Prolonged infections should be considered, such as viral infections (*e.g*. adenovirus) or “pertussis-like” infections [14, 16]. Prospective studies, carried out on the first line of care found 12% of cases involved Mycoplasma pneumoniae and between 20 and 37% involved Bordetella pertussis when serology was routinely performed for a prolonged cough [18, 19]. One study mentioned a longer cough time when a diagnosis of pertussis was proposed, or 118 days on average in this case (with 95% CI 82 to 154 days), 39 days of coughing being the average length when Mycoplasma pneumoniae was identified (with 95% CI 24 to 54 days) [20].

- For a cough lasting eight weeks or more, a chronic cough, asthma, rhinosinusitis, prolonged bacterial bronchitis, environmental agents and gastro-esophageal reflux are the main causes [6, 21, 22].

The level of evidence regarding diagnosis and treatment of a cough lasting more than eight weeks in a primary setting is low. The only study found in general practice is descriptive and was concerned with diagnoses made by the GP. It mentions ENT (ear-nose throat) etiologies infections, asthma, and gastroesophageal reflux as the principal etiologies [6]. Other epidemiological studies are performed in specialized care studies and the recommendations are based on these studies and based on expert opinions.

Furthermore, you will find below some controversies related to the etiologies of chronic cough. Among the main causes, asthma and prolonged bacterial bronchitis are commonly accepted etiologies [17, 23, 24]. Environmental factors, particularly exposure to tobacco smoke, are known to be associated with longer and more frequent episodes of coughing [17]. The postnasal drip is mentioned by the English guidelines [17] and our previous version [24], while others do not consider it, arguing that the phenotype of the cough is very different. It seems appropriate to us to keep postnasal drip as a possible diagnosis within a clinical practice guideline for the first line of care since above all we want to take account of the patients complaints. However, the cough’s pattern could be very heterogeneous. As regards gastroesophageal reflux, its role is controversial in regard to the etiology of isolated coughing, and antacids have shown no effect in resolving the development of a cough present concomitantly with reflux in young children [25]. Similarly for isolated non-specific coughing, one should be careful before making a diagnosis of asthma, which is only rarely a cause in the absence of wheezing or other suggestive signs [17].

### 1.4 Age Related Etiology

The recommendations of EBM PracticeNet mention that etiologies vary according to age [26].

Thus, besides frequent infections that can occur at any age, structural abnormalities of the airways should be mentioned, particularly in regard to infants.

Among preschool children, the incidence of asthma is increasing, and the inhalation of foreign bodies is more specific to this age group; in children of school age, psychogenic causes may also be mentioned.

## 2 AIMS OF GUIDELINES

These recommendations are intended to aid in the diagnosis and treatment of children attending a general practitioner with the complaint of a prolonged cough. More specifically, we aim to answer the questions raised by a group of experts in general practice to assist GP’s in their current practice.

## 3 METHODS

### 3.1 Definition of Clinical Questions

Our literature review was conducted to find answers to clinical questions drafted by the authors’ group and discussed in the Belgium general practice scientific group. The authors formulated seven clinical questions based on their own experience and their knowledge of the expectations of the target group. The questions were selected by the leading group responsible for primary care guideline development depending on their clinical relevance to general practitioners . The chosen clinical questions were the following:

1 ) What clinical approach is used to cope with a prolonged cough?
2 ) For what duration of a cough can an additional check be helpful in avoiding complications related to diseases that do not resolve themselves (bronchiectasis *etc*.)?
3 ) Is a prolonged wet cough more often complicated than a prolonged dry cough in the first line of care ?
4 ) Is there a benefit to treating a prolonged cough in terms of resolution of coughing episodes or reducing complications?
5 ) Are there any non-drug measures that are effective in reducing the duration or intensity of coughing episodes in children?
6 ) Are there effective medications for treating symptoms that reduce the duration or intensity of coughing episodes in children?
7 ) What are the risks of using cough medicines in children under and over six years of age?

## 3.2 Literature Search

These guidelines were produced following the “ADAPTE” procedure [27]. This adaptation involves three phases (set-up, adaptation and finalization), nine modules and 24 steps. After definition of the clinical questions, the adaptation phase involves searching for sources of clinical guidelines, assessing their content regarding the questions, assessing the quality and the coherence of available guidelines, assessing applicability and adaptation of recommendations to the target setting, a literature update, adaptation of the guidelines’ format, and implementation.

Firstly, practice guidelines were researched on the following websites: Guidelines International Networks (GIN), Centre Fédéral d’expertise des Soins de santé, National Guideline Clearinghouse, the National Institute for health Care and Excellence), Agence National de Sécurité du Médicament et des produits de santé, Canadian Association Medical Journal, Tripdatabase, Centre Belge Information Pharmaceutique), and the British Thoracic Society, and were selected in regard to the clinical questions

Five guidelines have been considered: the Belgian, British, Australian, Finnish and Nederland’s Guidelines [14, 17, 26, 28, 29]:

Secondly, two independent researchers have assessed their methodological quality regarding the AGREE instrument [30-32] (Appraisal of Guidelines for Research and Evaluation). The average scores of the two evaluators were noted, and discrepancies of more than two points were discussed.

Thirdly, to evaluate the contents of the selected practice guidelines, a matrix was created in a Microsoft Excel ™ spreadsheet showing the clinical issues. For each practice guide, the key messages with their level of evidence were extracted (if available) by clinical questions and placed in the matrix. The correspondence between the content(key messages) and the asked clinical questions was assessed and the level of evidence following the GRADE system [33] for each key message selected for our recommendation was analyzed . A pyramidal literature review was conducted: after looking for guidelines, we searched Cochrane and Medline from 2008 (the end date of the literature review of the BTS (British Thoracic Society) guidelines) until December 2014. For each clinical question the search strategy was determined taking into account PIPHO(Patient, Intervention, Professional, Outcome and Health care setting). A search was conducted of PubMed and Cochrane, with the following keywords: “chronic cough” “cough” [MeSH] and “children” [MeSH] and for each clinical question the search strategy was narrowed using “moist cough”, “wet cough”, “productive cough”, cough complication, “cough duration”, and “cough suppressant”

Articles meeting the PIPHO criteria were included. For patients, the pediatric population was defined as children aged 0 to 15, Research on coughing children with a previously diagnosed, prolonged or recurrent cough was excluded

Fourthly, the acceptability and applicability of the key messages in the Belgian context were checked by the authors’ group, and finally selective adaptation of relevant key messages was performed. For each of the key messages, we checked whether:

- The expertise was present and accessible, and thus usable in the Belgian situation.
- The conditions for application at organization level (*i.e*. daily practice.) were present to allow implementation in general medical practice.
- The key messages could be extrapolated to the target Belgian patients.

So that key messages were graded evenly and reproducibly in regard to their value, we translated their level of evidence / grade of recommendation given in the original population by following a GRADE system (Table **1**) when reviewing the primary literature upon which the recommendation was based.

The text of the guidelines was then reworked and refined following various meetings between the authors. It was submitted and discussed by methodological and content experts.

The text was reworked editorially and validated by the Belgian center for evidence-based medicine in September 2016.

## 4 RESULTS

### 4.1 Question 1: What Clinical Approach Should be Used to Treat a Prolonged Cough?

1. It is recommended to direct the medical history and physical examination towards searching for warning signs (Grade 1B) and towards the common etiologies depending on cough duration (recommendations of good practice).

#### 4.1.1 Explanation

Whatever the duration of the episode, general warning signs will be sought, such as respiratory distress, a stalled growth curve, fever and severe vomiting, and neurodevelopmental impairment. More specific signs such as thoracic deformation, respiratory or cardiac abnormal auscultation, chest pain, cyanosis, hemoptysis, digital clubbing, dysphagia, the risk context of tuberculosis or serious infections of the lower respiratory tract should be checked [15, 17, 34]. Neonatal onset and a family history of respiratory disease will also be evaluated. The diagnoses considered in these situations include an inhaled foreign body, immunodeficiency, congenital malformation, cystic fibrosis, pulmonary inhalation (related to a malformation, gastroesophageal reflux disease, a neuromuscular or neurodevelopmental disease), tuberculosis, or an interstitial disease [17]. As for the warning signs [15, 34], a study validated them in the context of children suffering with prolonged cough. This study, with a high prevalence of specific cough (88%), shows that in the absence of specific signs and symptoms (wheezing, crackles, asymmetry, on auscultation, abnormal cardiac auscultation, chest pain, dyspnea or tachypnea, chest deformity, clubbing, break in the growth curve, eating difficulty, hemoptysis, repeated severe infection, neuro developmental anomaly, daily wet cough, early neonatal onset, fever) and without indications following spirometry and chest X-ray, it is reasonable to exclude a cough requiring a specific treatment and to propose a ‘wait and see’ attitude. The negative likelihood ratio of all these factors combined is 0 (with a 95% CI of 0 to 0.03).

Orientation questions [17, 28] associated with the most common etiology are related to the onset of the cough, the cough’s quality, the cough evolution, associated symptoms cough triggers risk of allergy, the effect of the treatment, evaluation of environmental triggers, and vaccinations. The guiding questions come from the BTS guidelines and the first version of the Belgian guidelines. In accordance with the opinion of the experts consulted, the issue of vaccination was added. This is good practice in expert opinion. These signs and symptoms have not been specifically evaluated for prolonged cough.

### 4.2 Clinical Question 2: After What Duration of Cough is an Additional Assessment Useful to Avoid Complications?

2.1 For a cough persisting between four and eight weeks, indicators of a specific cause requiring investigation and / or particular treatment should be carefully sought (Grade 1C).

2.2 If the cough lasts more than eight weeks, chest radiography and spirometry (for children over six years) should be considered (Grade 2C).

2.3 Other diagnostic tests will depend on clinical suspicions (Grade 2C).

#### 4.2.1 A Cough Persisting Between Four and Eight Weeks

Evidence from observational studies suggested that within this range of time, cough is mostly due to prolonged infection or some specific infections, for example in 17% to 37% of cases, Bordetella pertussis is found [16, 18, 20, 26, 35]. The research into Mycoplasma pneumoniae, Chlamydia pneumoniae, and Bordetella pertussis, can allow a diagnosis to be made and the parents to be informed about the likely development of the cough. However, there is a lack of evidence about the effectiveness of a specific or symptomatic treatment in resolving the coughing episode [17, 36, 37]

Based on those studies and experts’ opinions, without signs or symptoms of a specific cause, an observation period of up to eight weeks from the development of the cough can be proposed [15, 17]. If there are warning signs or worsening of the cough in frequency or intensity, a chest X-ray will be considered, followed by other examinations specific to the suspected etiology. A high prevalence study [15] of specific cough, *i.e*. those who require treatment, shows that, in the absence of specific pointers and suggestive elements obtained from spirometry and chest X-ray, we can reasonably exclude a cough requiring specific treatment in children complaining of a prolonged cough, and can adopt a ‘wait and see’ attitude. The negative likelihood ratio of these combined factors is 0 (with 95% CI of 0 to 0.03) [15].

#### 4.2.2 A Cough Lasting Eight Weeks, or a Chronic Cough

A chest X-ray is suggested by various guidelines if the cough does not improve. The time limit varies according to the guidelines, with a maximum waiting period of eight weeks [14, 17, 23]. The purpose of this X-ray is to rule out an infection or an inhaled foreign body, and to indicate other additional tests if necessary.

A chest X-ray and spirometry (with reversibility) were of interest for identifying a specific cause of coughing during a study conducted on the third line of care [21]. This study showed that in regard to the discovery of an anomaly, the chest X-ray has a sensitivity of 70%, a specificity of 58% and a positive likelihood ratio of 1.66 for a specific cause of coughing. The extent to which these findings can be extrapolated to primary care is difficult to judge. Another recent study [15] conducted in hospitals found the following values ​​for an abnormality in the chest X-ray: positive likelihood ratio: infinite; negative likelihood ratio of 0.8 (with a 95% CI of 0.75 to 0.85).

The recommendations also propose carrying out spirometry in children over five years with a cough lasting eight weeks or more [17, 23]. If there is suspicion of asthma, spirometry (conducted from six years) has an infinite positive likelihood ratio and a negative likelihood ratio of 0.13 (with a 95% CI of 0.09 to 0 .17) [15].

In chronic wet cough, an analysis using a sputum culture can be helpful. However, this may be difficult to achieve with young children and may require the intervention of an experienced physiotherapist [17].

In children with no evidence of a specific issue, other investigations are rarely needed [23].

A clinical trial conducted on the third line of care suggests the effectiveness on the resolution of the cough of using a decision algorithm based on a chest X-ray, systematic spirometry, and the evaluation of warning signs [34].

### 4.3 Question 3: Is a Wet Cough Predictive of Complications in the First Line of Care?

There is a lack of data in the literature regarding the predictive value of the characteristics of a wet cough in regard to the first line of care in terms of complications.

3.1. In the case of a cough of more than eight weeks, it is recommended to investigate the presence of bronchiectasis (Grade 1C).

3.2. Faced with a cough of more than eight weeks, it is suggested to consider protracted bacterial bronchitis (Grade 1B)

The wet characteristic of a child’s cough as determined by the clinician or parents has good validity in terms of production found by bronchoscopy (K=0.75 95% CI 0.58-0,93 for clinician assessment of wet cough and bronchoscopic secretion grade 4) [38]. A chronic cough in children attending a specialist consultation is associated with prolonged bacterial bronchial infection, of which a complication may be bronchiectasis [38]. The predominant clinical signs of bronchiectasis are prolonged cough and auscultatory asymmetry [22, 39]. The wet character of the cough has proved to be the most predictive factor of a specific cough (OR = 9.34 with a 95% CI of 3.49 to 25.03) [21, 39], and the positive likelihood ratio for a specific cause of coughing is 26.15 (with a 95% CI of 3.77 to 181.48). This study included children coughing for more than three weeks. In fact, the average duration of cough was 15 weeks.

The duration of cough (especially when it lasts more than three months) is correlated with a radiological score indicating more severe bronchial involvement, which for children is referred to as a prolonged cough [40].

Another study, also in a specialized environment, showed that a wet cough is associated more frequently than a dry cough with a bacterial or viral infection of the lower respiratory tract during bronchoalveolar lavage [41]. If the first question that arises concerns the choice between diligent waiting and a more proactive attitude, the wet character of the cough is the factor most associated with a specific cause [15].

According to clinical practice guidelines, other less common pathologies [42] should be considered in the case of a prolonged cough, such as the presence of bronchiectasis or mucociliary dyskinesia. The diagnosis of prolonged bacterial bronchitis should be suggested for a prolonged cough without other warning signs of underlying causes and with a positive sputum culture [17], and which resolves after antibiotic treatment (lasting two weeks) [23].

### 4.4 Question 4: Is There a Benefit to Treating Prolonged Cough in Terms of Resolution of the Coughing Episode or Reducing Complications?

4.1. An antibiotic is recommended for prolonged wet cough (over eight weeks) if prolonged bacterial bronchitis is suspected, after exclusion of other underlying causes (Grade 1B).

It is important to first of all determine the etiology of the cough and carefully exclude a specific underlying cause. If a specific etiology is diagnosed, this should be treated first [17].

If the diagnosis is suggestive of bacterial bronchitis, antibiotic treatment will be considered.

A study published in 2012 showed the effectiveness, in terms of cough reduction, of antibiotic treatment. Another study published in 2014 showed the effectiveness of a decision-making algorithm if used early and including the factor of loose cough, on the resolution of the coughing episode. Both studies were conducted on a population of children with chronic cough, selected from the third line of care. The selection criterion was a cough of more than three weeks, and the cough term average was in fact 15 weeks [15, 34, 42, 43]. A study published in 2012 established the efficacy of amoxicillin-clavulanic acid on prolonged wet coughs in children enrolled in specialized outpatient consultation with NNT 4 (with a 95% yield of 3 to 5) [43]. This study supports the results of an earlier meta-analysis (n = 140) in which antibiotics reduced the number of children not cured during the monitoring visit with an OR of 0.13 (with a 95% CI of 0, 06 to 0.32) [39].

Studies on the first line of care are also needed, given the wide diagnostic spectrum.

Studies conducted on the third line of care thus show the relationship between the wet characteristic of a cough, the presence of secretions, and bronchial infection on the one hand, and the resolution of the cough after treatment with amoxicillin-clavulanic acid on the other. There are no studies on the first line of care. There are also no studies showing the impact of treatment of prolonged wet cough on complications such as the emergence of bronchiectasis. Given the context of the evolution of antimicrobial resistance and the required duration of antibiotic therapy, prior antibiotic susceptibility testing is preferable to blind treatment.

Physical therapy may also be useful in the case of prolonged wet cough without any previously mentioned underlying etiology [17].

### 4.5 Question 5: Are There Any Non-Drug Measures that are Effective in Reducing the Duration or Intensity of Coughing Episodes in Children?

An association was found between the presence of tobacco, moisture in the house, gas or coal stoves, heating with wood or kerosene and the frequency and / or increased duration of cough.

5.1. In the case of a prolonged cough, it is suggested initially to avoid irritants (tobacco, mold, stoves *etc*.) (Grade 1C).

5.2. It is recommended to take into account the concerns of parents and inform them about the natural development of a cough (Recommendation of good clinical practice).

The recommendations suggest avoiding irritants [17, 23]. Several studies show, in fact, the link between environmental factors and episodes of longer or more frequent cough (24). The literature review did not identify intervention studies in this area [10].

For a child, a prolonged cough can be an emotional burden for himself and for his parents; it affects the quality of life and sleep as well as schooling [10]. Stress will be reduced when the cough episodes are resolved [10, 34]. There is no literature on the effect of information or a comprehensive approach in the specific context of chronic cough. In the case of acute cough, information given to parents beforehand has an effect on consultation rates [44, 45]. Moreover, the implications for the child, and taking account of the expectations of parents, influence the rate of consultation and antibiotic prescription. The recommendations therefore advocate recognizing the expectations and fears of the parents and their children, and informing them about the normal progression of symptoms [23].

Treatments administered to treat cough may act in different ways, according to studies. The following may occur: spontaneous improvement of self-limiting disease, physiological effects of the excipients (mucociliary activation, salivation), true placebo effect, and finally the impact of the molecule herself. This may explain why an effect is generally observed in both arms of clinical trials, including in patients on a sweet syrup or placebo [17, 46-48].

The placebo effect as well as the physiological effect of sweet substances are supported by a good quality essay suggesting the superior efficacy of honey versus no treatment and versus diphenhydramine, and an efficacy equivalent to dextromethorphan on the intensity of the cough [49, 50]. Honey could be effective and safe, though it should be used with caution for children less than one year old, since, because of the immaturity of their intestinal mucosa, they are more susceptible to botulism spores that could be contained in the honey [50]. A trial published in 2014 also showed a significant effect on cough scores of placebo (grape juice) versus a pure ‘wait and see’ approach [51].

### 4.6 Question 6: Is There an Effective Drug Symptomatic Treatment to Reduce the Duration or Intensity of Coughing Episodes in Children?

6.1. In the absence of clinical signs of a specific etiology of a cough, no drug can be recommended because of the lack of evidence of any drug’s superiority compared to placebo (Grade 1B).

Without red flags or signs of common disease, in children suffering from isolated non-specific cough, it is not recommended and does not seem beneficial to provide empirical anti-asthma [51-53] or anti-reflux treatments [25], or treatment for allergic rhinitis [17].

For some molecules, clinical trials have been conducted in children with acute cough (dextromethorpan [54-56], codeine, antihistamine [57]) or chronic non-specific cough(ß-agonist [57], beclometasone [52]) and these have failed to demonstrate the superior efficacy of the molecule versus placebo. For other molecules (mucolytics [58, 59], *i.e*. expectorants, anticholinergics [60], leukotriene receptor antagonists [61], antibiotics [62]), there is a lack of quality clinical trials showing any effectiveness.

### 4.7 Question 7: What are the Risks of Using Symptomatic Cough Treatment in Children Under Six / Over Six Years?

All antitussives are contra-indicated in children under six because their risk/benefit balance is unfavorable (Grade 1B).

Between six and 12 years, their use is not recommended (Grade 2B).

All mucolytics and expectorants are contra-indicated in children under two years and guaifenesin is contra-indicated below six years (Grade 1B).

Between six and 12 years, mucolytics and expectorants are not recommended (Grade 2C).

#### 4.7.1 Terpene Derivatives

These are aromatic organic derivatives. They are found in the essential oils of pine, thyme, eucalyptus, and niaouli. They are also found to some degree in camphor, menthol, terpin and terpineol. The fact that they are not registered as drugs but as dietary supplements also makes them qualitatively inconsistent. There is no longer any medicine registered based on terpene derivatives (see below for adverse effects and contra-indications of pholcodine). On the other hand, they can be found in dietary supplements or health food stores.

Their side effects are mainly neurological (convulsions) and may occur following topical application. Ingestion of 15ml of eucalyptus essential oil can be fatal (6).Menthol and eucalyptol may cause laryngospasm [24].

Terpene derivatives are contra-indicated in children under 30 months and in the case of history of febrile convulsions or anorectal lesions (Grade 1B).

#### 4.7.2 Expectorants, Mucolytics

Following a decision of the FAMHP (Federal Agency for Medicines and Health Products) acetylcysteine [54, 59] is contra-indicated below two years of age because it can cause paradoxal bronchorrhoea. The other side effects are mainly gastrointestinal: nausea, vomiting, diarrhea.

Carbocisteine ​​presents mainly gastrointestinal adverse effects and headache [6].

The European medical agency has recently reassessed the benefit/risk of bromhexine and its metabolite ambroxol in pediatrics, and warns that hypersensitivity reactions and skin reactions can occur, which are rare but sometimes severe. Moreover, the effectiveness of these drugs is limited [55].

#### 4.7.3 Centrally Acting Antitussives

Codeine and its derivatives (dihydrocodeine, ethylmorphine and thebacon) have side effects of drowsiness, constipation, respiratory depression, nausea, edema and pruritus [6]. Codeine is partly metabolized to morphine, and can cause addiction if administration is prolonged. All drugs containing codeine and its derivatives are issued only on prescription [24].

*Dextromethorphan* seems safer than codeine, but can cause excitement, confusion, respiratory depression, dizziness and gastrointestinal disorders [24].

*Pholcodine* has a half-life of over 50 hours and must be used with great caution if taken repeatedly. A case of fatal toxicity has been described in children. Its side effects are digestive problems and headaches as well as somnolence [24].

*Cloperastine* has anticholinergic effects (dry mouth) [24].

#### 4.7.4 Antihistamines and Decongestants

All these special products, usually containing multiple ingredients, are sold without prescription. However, some have side effects that can be serious.

Antihistamines can cause drowsiness, gastrointestinal disorders and, in small children, confusion and / or cardiorespiratory failure.

Side effects of vasoconstrictors used as oral decongestants are elevated blood pressure, restlessness or lethargy. Vasoconstrictors used as local decongestants in resolving troublesome ENT symptoms can cause adverse neurological, psychiatric and cardiovascular effects [6].

## CONCLUSION

More research is needed to provide evidence on the clinical pathway on prolonged cough for primary care. The main recommendations are as follow:

- It is recommended to direct the medical history and physical examination towards searching for warning signs (Grade 1B) and towards the common etiologies depending on cough duration (recommendations of good practice).
- For a cough persisting between four and eight weeks, indicators of a specific cause requiring investigation and / or particular treatment should be carefully sought (Grade 1C).
- If the cough lasts more than eight weeks, chest radiography and spirometry (for children over six years) should be considered (Grade 2C).
- In the case of a wet cough of more than eight weeks, it is recommended to investigate the presence of bronchiectasis (Grade 1C).
- Faced with a wet cough of more than eight weeks, it is suggested to consider protracted bacterial bronchitis (Grade 1B).
- In the case of a prolonged cough, it is suggested initially to avoid irritants (tobacco, mold, stoves etc.) (Grade 1C).An antibiotic is recommended for prolonged wet cough (over eight weeks) if prolonged bacterial bronchitis is suspected, after exclusion of other underlying causes (Grade 1B).
- It is recommended to take into account the concerns of parents and inform them about the natural development of a cough (Recommendation of good clinical practice).
- In the absence of clinical signs of a specific etiology of a cough, no drug can be recommended because of the lack of evidence of any drug’s superiority compared to placebo (Grade 1B). All antitussives are contra-indicated in children under six because their risk/benefit balance is unfavorable (Grade 1B). All mucolytics and expectorants are contra-indicated in children under two years and guaifenesin is contra-indicated below six years (Grade 1B).

## Figures and Tables

**Table 1:** Grade used in the recommendation.

**Grade of recommendation**	**Clarity of risk/benefit**	**Quality of supporting evidence**	**Implications**
**1A.** Strong recommendation, high quality evidence	Benefits clearly outweigh risk and burdens.	Consistent evidence from well performed randomized, controlled trials or overwhelming evidence of some other form.	Strong recommendation, can apply to most patients in most circumstances without reservation.
**1B.** Strong recommendation, moderate quality evidence	Benefits clearly outweigh risk and burdens.	Evidence from randomized, controlled trials with limitations or very strong evidence of some other research design.	Strong recommendation and applies to most patients.
**1C.** Strong recommendation, low quality evidence	Benefits appear to outweigh risk and burdens.	Evidence from observational studies, unsystematic clinical experience, or case reports.	Strong recommendation, but could change if new evidence appears.
**2A.** Weak recommendation, high quality evidence	Benefits closely balanced with risks and burdens.	Consistent evidence from well performed randomized, controlled trials or overwhelming evidence of some other form.	Weak recommendation, best action may differ depending on circumstances or patients or societal values.
**2B.** Weak recommendation, moderate quality evidence	Benefits closely balanced with risks and burdens.	Evidence from randomized, controlled trials with limitations or very strong evidence of some other research design.	Weak recommendation, alternative approaches likely to be better for some patients under some circumstances.
**2C.** Weak recommendation, low quality evidence	Uncertainty in the estimates of benefits, risks, and burdens; benefits may be closely balanced with risks and burdens.	Evidence from observational studies or case reports.	Very weak recommendation; other alternatives may be equally reasonable.
